# Books and Magazines

**Published:** 1906-01

**Authors:** 


### Books and Magazines.

FOOD IN HEALTH AND DISEASE. By Robert F. Williams, M. A., M. D., Professor of Principles and Practice of Medicine in the Medical College of Virginia, Richmond.

The volume will be a convenient 12mo of about 350 pages. Its price has not yet been fixed, but it will probably be about $2, net, delivered to any address.

It is divided for convenience, into two parts: Part I. dealing with Food in Health, and Part II. with Food in Disease.

In Part I. the needs of the body for different kinds of foods and the manner in which they are utilized are explained. The principles of cooking foods and detailed descriptions of the different articles of food in common use are given, with chapters on the proper nutriment of infants, children, adults, and the aged.

Part II. deals with the variations from the normal diet in health, necessitated by the more common diseases, and includes a chapter on general methods to be observed in feeding the sick, as well as the special directions for nourishment in diesases of different kinds.

There exists today a need for a small practical book on foods and how they should be used, which will give the facts, as known today, in a brief and clear manner, with the fewest possible technical terms.

The importance of a work of this kind, which is simple enough for a child to read and yet absolutely trustworthy and based upon the scientific achievements of accepted leading authorities, is obvious. Such books are in line with the best principles of hygiene and make for the betterment of the present as well as future generations.

---

MINOR AND OPERATIVE SURGERY, INCLUDING BANDAGING. By Henry R. Wharton, M. D., Professor of Clinical Surgery in the Woman's College; Surgeon to the Presbyterian Hospital, Philadelphia, etc. New (6th) edition, enlarged and thoroughly revised. In one 12mo volume of 642 pages, with 532 illustrations. Cloth, $3, net. Lea Brothers & Co., Publishers, Philadelphia and New York, 1905.

Dr. Wharton's work has for many years been the accepted authority in its field, and justly so.

In its many revisions its scope has been gradually broadened in response to the suggestions of its many readers, so that in the present edition it really covers all except what might be termed capital surgery. The increased attention which is given in the medical colleges to operative procedures on the cadaver and the importance of this method of instruction have led the author to include those operations which can be advantageously taught in this way, such as Ligation of Arteries, Amputations, Excision of Joints, Operations upon the Nerves and Tendons, Intestinal Anastomosis, etc., etc. The various bandages and surgical dressings are clearly described, and illustrated by numerous engravings. Particularly will the helpfulness of the illustrations be shown in the section on bandaging where almost every variety of bandage or dressing is explained in detail, and shown with such clearness that a novice could scarcely fail in the application of one.

The importance of sepsis and antisepsis has received full consideration, and Surgical Bacteriology is covered in a special chapter.

